# Supplementary material for: The genome sequence of Gwynne’s mining bee, Andrena bicolor Fabricius, 1775
Source: Wellcome Open Res. 2024 Mar 8;9:140. [Version 1] doi: 10.12688/wellcomeopenres.21104.1 (PMC11153990; doi:10.12688/wellcomeopenres.21104.1)
Supplement: Peer review history

# Reviewer report: Reviewer response for version 1

**DOI:** 10.21956/wellcomeopenres.23344.r82119

**Published:** 2024-06-05

**Authors:** Jan Veenstra

**Affiliations:** 1 INCIA, UMR 5287, CNRS, University of Bordeaux, Pessac, France

It is nice to see Steven Falk being the first author on this manuscript. It illustrates the good collaboration between field and molecular biologists. The technology of high quality DNA extraction, sequencing and subsequent genome assembly has improved so much and the genome assemblies that are produced in almost industrial quantities by the Welcome Sanger Institute are so high quality that there is nothing to criticize. Fortunately, referee reports now only need a minimum of 50 words.

 There are two very general comments I do wish to make, although they have no direct bearing on this manuscript. First, there are several insect genome assemblies that are of very poor quality, yet concern important pests and disease vectors. Although I am happy to see a perfect genome assembly for Andrena bicolor, I can't help wondering whether some of the effort by the Welcome Institute might not be directed toward some of these species.

 A second comment concerns the Busco sequences. In this species, as in most that were sequenced by the Welcome institute the percentage of identifies Busco sequences is again very high. Given the very high quality of this and many other genome assemblies, one wonders which are the Busco sequences that are missing. Are they genuinely absent from these species, and if so which proteins do they code for and is there a pattern of genuinely absent sequences. If we wanted to understand this, it might perhaps be useful to have a list of absent and present Busco sequences, so it could be worthwhile to consider to add an additional data set of present and absent Busco sequences to each sequence assembly.

Are sufficient details of methods and materials provided to allow replication by others?

Yes

Is the rationale for creating the dataset(s) clearly described?

Yes

Are the datasets clearly presented in a useable and accessible format?

Yes

Are the protocols appropriate and is the work technically sound?

Yes

Reviewer Expertise:

Insect Neuropeptides

I confirm that I have read this submission and believe that I have an appropriate level of expertise to confirm that it is of an acceptable scientific standard.

# Reviewer report: Reviewer response for version 1

**DOI:** 10.21956/wellcomeopenres.23344.r79193

**Published:** 2024-05-22

**Authors:** Natalia Araujo

**Affiliations:** 1 Unit of Evolutionary Biology & Ecology –, Universite Libre de Bruxelles, Brussels, Brussels, Belgium

The article reports the assemblies of the nuclear and mitochondrial genomes of *Andrena bicolor* a mining bee of western palearctic distribution, no annotation of the reference genome is provided. The nuclear genome spanned 351.7 Mb across 399 contigs, mostly comprised within 5 large scaffolds. The authors used the Darwin Tree of Life pipeline to generate this assembly, a largely accepted methodology, so I have no major concerns about this work. A few minor suggestions for improving the report are:

  

1. I don’t work with *Andrena* bees, so I was surprised about the small chromosome numbers reported and their large sizes (up to 65.38 Mb). Looking at the Hi-C map (Figure 5), it was not so clear to me why during manual curation scaffolds 1, 2, 4, and 5 were not split as they show low support regions that could represent possible missjoints. Then, I searched for the expected chromosome numbers in *Andrena* and I saw these bees have an expected small chromosome number. I thus concluded that the authors, based on this information, considered these regions could be explained by specific chromosome conformations in this species. This is reasonable, but I suggest it be clarified further in the text. Is the chromosome number of this species confirmed by other methods? Did the authors consider this information during scaffolding? I understand these are pseudochromosomes, but it would be nice to know how their number fits within the species or genus expected numbers.  
2. In the abstract and the Genome sequence report sections, it says the genome was assembled from a single female. Actually, the Pacbio sequencing used a single female but the Hi-C data was generated using a male. Thus, two specimens were used to produce this assembly and not only one as stated. This should be corrected.
3. The annotation of the mitochondrial genome is mentioned but there is no information about it. Did it comprise all genes? Where it can be assessed?
4. In the MitoHiFi description, in the methods section, the authors describe that this pipeline may use two approaches for annotation (MitoFinder or MITOS) which one did they use for the current assembly?

Are sufficient details of methods and materials provided to allow replication by others?

Yes

Is the rationale for creating the dataset(s) clearly described?

Yes

Are the datasets clearly presented in a useable and accessible format?

Yes

Are the protocols appropriate and is the work technically sound?

Yes

Reviewer Expertise:

bee genomics and evolution, bioinformatics.

I confirm that I have read this submission and believe that I have an appropriate level of expertise to confirm that it is of an acceptable scientific standard.
